# The GIMAP Family Proteins: An Incomplete Puzzle

**DOI:** 10.3389/fimmu.2021.679739

**Published:** 2021-05-31

**Authors:** Marc-André Limoges, Maryse Cloutier, Madhuparna Nandi, Subburaj Ilangumaran, Sheela Ramanathan

**Affiliations:** Department of Immunology and Cell Biology, Faculty of Medicine and Health Sciences, Université de Sherbrooke and CRCHUS, Sherbrooke, QC, Canada

**Keywords:** GIMAP5, gimap, lymphopenia, AIG domain, T lymphocyte, B cells

## Abstract

**Overview**: Long-term survival of T lymphocytes in quiescent state is essential to maintain their cell numbers in secondary lymphoid organs and in peripheral circulation. In the BioBreeding diabetes-prone strain of rats (BB-DP), loss of functional GIMAP5 (GTPase of the immune associated nucleotide binding protein 5) results in profound peripheral T lymphopenia. This discovery heralded the identification of a new family of proteins initially called Immune-associated nucleotide binding protein (IAN) family. In this review we will use ‘GIMAP’ to refer to this family of proteins. Recent studies suggest that GIMAP proteins may interact with each other and also be involved in the movement of the cellular cargo along the cytoskeletal network. Here we will summarize the current knowledge on the characteristics and functions of GIMAP family of proteins.

## Introduction

In the BioBreeding diabetes-prone strain of rats (BB-DP), the recessive *lyp* mutation causes a profound loss of T lymphocytes in secondary lymphoid organs (1). Positional cloning of the gene responsible for the lymphopenic phenotype in the BB-DP rats independently by two groups led to the discovery of a family of proteins that are conserved in vertebrates (2, 3). The *lyp* allele arises from a frame shift mutation within the GTPase domain of the immune associated nucleotide binding protein 5 (*Gimap5*) gene, resulting in a truncated protein lacking 223 amino acids at the C-terminus (2, 3). GIMAP5 is a member of the GIMAP family that are implicated in immune functions in mammals (4). Initially this family of proteins was named IAN, for ‘immune associated nucleotide binding’ proteins, as they were predominantly expressed in the cells of the hematopoietic system and contained domains that can bind to GDP/GTP. In this review we will summarize our current knowledge of the structure and functions of GIMAP proteins, many of which are implicated in the development and maintenance of lymphocytes (**Table 1**). All *Gimap* genes are clustered within a short locus in the genome. The human *GIMAP* cluster, spanning about 500kb on chromosome 7, contains seven functional genes and one pseudogene (4). In mice and rats the *Gimap* genes are present as a tight cluster within a 150kb region on chromosome 6 and 4, respectively (2, 3). *Gimap*-like genes have been identified in angiosperms, corals, nematodes and in snails wherein they are implicated in protection from infections (56–59). The observed homology between GIMAP proteins and the plant avrRpt2 induced gene 1 (AIG1) might have resulted from convergent evolution of the AIG1 domain (4, 60). In fact, initial homology searches identified *Arpt1* in *Arabhdiposis thaliana* (61) as the closest homolog of the mammalian *Gimap* family proteins.

**Table 1:** Phenotype of deficiency in *GIMAP* genes.

Gene/Protein localization	mice/rats	Humans	References
*Gimap5* Lysosomes, vesicles	**Rats:** Normal T cell development; reduced T cell export; peripheral T lymphopenia; Survival defects in naïve resting T cells; normal B and NK cells; T cell-mediated autoimmunity dependent on the genetic background; Normal life span; spontaneous activation of the AKT signaling pathway **Mice:** Deficiency in T, B and NK cells; survival defects in lymphocytes; exhaustion of HSC; hepatic extramedullary hematopoiesis; reduced life span; spontaneous activation of AKT signaling In another independent knockout mouse line, no defect was observed	T and NK defects; splenomegaly and lymphadenopathy; spontaneous activation of the AKT pathway; responsive to rapamycin treatment; replicative senescence in T cells.	Rats: (1–3, 5–34). Mice: (35–41). Humans: (40, 42).
*Gimap1* Golgi apparatus	Reduced survival of T and B cells; loss of mitochondrial potential and oxygen consumption	GIMAP1 expression is increased in DLBCL lymphomas	(33, 43)
*Gimap2* Lipid droplets	absent	Not known	(44–46)
*Gimap3* Endoplasmic reticulum	Pseudogene in rats In mice GIMAP3 regulates the segregation of mitochondrial DNA	Pseudogene in humans	(47, 48)
*Gimap4* Cytosolic	Required for the transition of T cells from apoptotic to dead cells	Associated with cytoskeleton, movement of vesicles and secretion of cytokines	(49, 50)
*Gimap6* Autophagosomes	Reduced T and B cell numbers; Increased sensitivity to apoptosis	Increased sensitivity to apoptosis	Mice: (51–53). Humans: (54).
*Gimap7* Cytosolic	Not known	Not known	(44–46)
*Gimap8*	Reduction in recirculating B cells		(55)

^1^Among the GIMAP genes, only GIMAP5 appears to affect the longevity of mice and possibly, humans.

**Figure 1:**
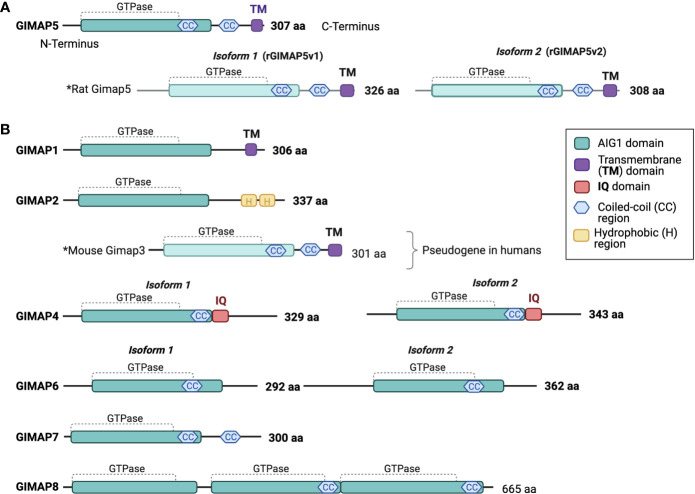
Human GIMAP family protein structure. Predicted structural domains of human GIMAP family members. **(A)** The 307-amino acid long human GIMAP5 protein contains the GIMAP GTPase domain, coiled-coil (CC) regions and a C-terminal transmembrane (TM) domain. Rat GIMAP5 protein is found in two isoforms that differ in length (GIMAP5v1 and GIMAP5v2). Both retain the CC regions and the TM domain. **(B)** Predicted structures of other human GIMAP family proteins and mouse GIMAP3, as the latter is a pseudogene in humans. The calmodulin interaction domain (IQ) is unique to both GIMAP4 isoforms. The drawings are not to scale.

The GIMAP family consists of putative small GTP-or/and ATP binding proteins that are conserved among vertebrates (**Figure 1**). Among the GIMAP family proteins, sequence similarity is restricted to the N-terminus, which contains a guanine nucleotide-binding domain (4, 62). All GIMAP family members harbor the AIG1 domain containing a GTP-binding motif, that is referred to as GIMAP GTPase domain comprised of the five G motifs G1, G2, G3, G4, and G5, which are involved in nucleotide binding. It is thought that the GIMAP GTPase domains may be functional and that their activity may be controlled by homo/hetero dimerization (44–46, 63). However, only some GIMAPs have been shown to bind GDP/GTP or to hydrolyze GTP. GIMAP4 is the first member reported to bind GDP and GTP and exhibit GTPase activity (63). Further studies have shown that GIMAP2 and GIMAP5 can bind GTP with high affinity but cannot hydrolyze it on their own (44). GIMAP7 can stimulate its own GTPase activity and promote GTP hydrolysis by GIMAP2 (44). Besides the GTPase domain, all GIMAP proteins contain a helical segment that folds back on to the GTPase domain and may mediate interaction with partner proteins. In addition, GIMAP1, GIMAP3, and GIMAP5 contain a transmembrane hydrophobic domain at the C-terminus that have been shown to mediate membrane anchoring and target them to intracellular organelles (64). In the next sections each of the GIMAP family member is discussed in detail, starting with the founding member GIMAP5.

## GIMAP5

### Mutation in *Gimap5* Is Associated With T Lymphopenia

A spontaneous mutation in an outbred colony of Wistar rats was associated with the development of autoimmune type 1 diabetes (T1D) (5). Incidentally these rats were also lymphopenic. Further inbreeding resulted in the establishment of the strain of BB-DP rats (1, 6–8). Genetic studies mapped the lymphopenia phenotype to the *lyp* locus on chromosome 4 (9). In 2002, two groups independently identified a frameshift mutation within the *Gimap5* gene as being responsible for this lymphopenic phenotype (2, 3). In BB-DP rats, lymphopenia is restricted to the T cell compartment with a 5-10 fold reduction in the number of mature T cells in secondary lymphoid organs (10). This lymphopenia is more severe in the CD8^+^ T cell compartment than in the CD4^+^ T cell compartment (11, 12). Thus, most studies characterizing the function of GIMAP5 in T cells have been carried out on the CD4^+^ T cell subset. Mature CD4^+^ T cells, characterized by the expression of the RT6 marker in the rats, were almost completely absent in the secondary lymphoid organs of BB-DP rats (13, 14). *In vitro* studies have shown that the few surviving T cells in BB-DP rats are not fully functional. Purified BB-DP rat CD4^+^ T cells stimulated with mitogens or anti-CD3/CD8 antibodies displayed impaired proliferative response (15). *In vivo*, the decreased functionality of the BB-DP rat T cells was attributed to the increased nitric oxide (NO) production by macrophages (16). Bone-marrow chimeras showed that the increased NO production by macrophages of the BB-DP rats was secondary to the T lymphopenia, as correcting the latter decreased NO production (15). In addition, the *lyp* mutation affects the development of regulatory T cells, γδ T cells and intra-epithelial lymphocytes (iIELs) in the intestinal mucosa (17, 18). The development and survival of NK cells and B lymphocytes are not affected by the *lyp* mutation in BB-DP rats (genotype: *Gimap5^lyp/lyp^*) (1). An important paradox in BB-DP rats that aroused much scientific investigation in the 80s and 90s was the T cell dependency of autoimmune T1D development despite severe lymphopenia. Depletion of the few CD4^+^ or CD8^+^ T cells, but not NK cells, prevented T1D development in BB-DP rats (19, 20), indicating that the remaining lymphocytes are the crucial mediators of disease.

### Phenotype of T Cells in the Absence of Functional GIMAP5

During T cell development, hematopoietic precursors with restricted multipotency enter the thymic cortex from the bone marrow and undergo a series of developmental changes that are demarcated by specific phenotypic characteristics associated with their commitment towards the T cell lineage (65). Until the rearrangement of the T cell antigen receptor (TCR) genes, thymocytes do not express CD4 or CD8 co-receptors and are referred to as double negative (DN) thymocytes. TCR rearrangement upregulates the expression of both CD4 and CD8 co-receptors, allowing these double positive (DP) cells to undergo positive selection (66, 67). Subsequent to TCR signaling, DP cells lose one of the two co-receptors, depending on their ability to recognize MHC class I or class II molecules, and become CD4 or CD8 single positive (SP) cells that transit to the thymic medulla where T cells with high affinity to self-antigens are deleted by the negative selection process. The surviving SP thymocytes undergo further maturation before exiting to the periphery as recent thymic emigrants (RTE). RTE undergo additional maturation in the periphery to become long-lived naïve T cells. Naïve T cell survival is maintained in the periphery by constant low-level interactions with the self MHC:peptide complexes (MHC:p) (68). Upon encounter with the cognate antigen, the TCR-stimulated T cell clones undergo proliferative expansion and initiate the adaptive immune response. Some of the antigen-specific T cells undergo reprogramming to become long-lived memory T cells that constantly patrol the tissues. Both naïve and memory T cells rely on cues from TCR- MHC:p interactions and cytokines such as IL-7 and IL-15 for their survival and homeostasis in the periphery.

Even though the proportion of DN, DP and SP subsets are comparable between control and BB-DP rats, the reduced thymic output in BB-DP rats (1, 21, 22) suggested that the lymphopenic phenotype caused by *Gimap5^lyp/lyp^* genotype manifests during the later stages of T cell development in the thymus. In line with this observation, transcripts for *Gimap5* are detected at higher levels starting from DP stage of T cell development in normal rats (23). Some groups have observed a decrease in the frequency of immature CD8^+^ SP thymocytes (24–26). Nevertheless, SP thymocytes from *Gimap5^lyp/lyp^* rats undergo accelerated apoptosis *in vitro* (1, 25–27).

Homeostatic expansion of T cells present in the periphery can compensate for reduced thymic export and can almost fully restore the T cell numbers under conditions of lymphopenia (69). However, the peripheral T lymphopenia in the *Gimap5^lyp/lyp^* rats suggests that the homeostatic expansion may also be compromised by GIMAP5 deficiency or, alternately, that the expanding cells are unable to survive and persist in the secondary lymphoid organs. To address this issue, we thymectomized control and *Gimap5^lyp/lyp^* rats and labeled the cycling cells with bromodeoxyuridine (27). Whereas only 5-10% T cells incorporated the label in control rats with a full T cell compartment, almost 100% of the T cells in *Gimap5^lyp/lyp^* rats had incorporated BrDU during the same period. Despite this increased T cell cycling in the periphery, *Gimap5^lyp/lyp^* rats fail to restore their T cells numbers in the periphery. Follow up of the BrDU-labeled cells during the chase period suggested that most of them were lost from the secondary lymphoid organs in *Gimap5^lyp/lyp^* rats while they were present in control rats. The complete loss of the BrDU-labeled cells from the secondary lymphoid organs of *Gimap5^lyp/lyp^* rats indicated that the progeny of the cycling cells was unable to persist and survive and that the homeostatic pressure maintains the few surviving cells in a perpetual cycling phase. Reconstitution of the lymphopenic BB-DP rats with splenocytes from syngenic, diabetes-resistant (BB-DR) rats that carry the wildtype *Gimap5* allele eliminated the cycling of endogenous BB-DP T cells that were eventually lost from the periphery.

Paradoxically, the few cells that persist in the secondary organs of *Gimap5^lyp/lyp^* rats have been shown to be activated by their cognate antigen and incorporated into the pool of recycling cells (27). As there are no TCR transgenics available for rats, the antigen specificity of the peripheral T cell pool was assessed using allogenic T cells from Wistar Furth (WF) background as lymphopenic BB-DP rats could reject allogenic T cells (27). To determine whether antigen-reactive cells were inducted into the pool of recirculating cells, the BB-DP rats were thymectomized following the rejection of WF T cells. One month after thymectomy, the antigen-exposed rats were still capable of rejecting the allogenic T cells. However, in the absence of prior exposure, thymectomized rats were unable to eliminate the allogenic cells. Additional experiments showed that RTEs had a narrow window of one week after thymic exit in order to be ‘rescued’ by TCR stimulation. These experiments helped resolve the paradox of T cell mediated autoimmunity in BB-DP rats. In an appropriate genetic background these cycling *Gimap5^lyp/lyp^* T cells recognize self-antigens and induce autoimmune diseases. For example, in the BB-DP rats these cycling T cells recognize islet antigens and induce T1D. In the PVG background the *lyp* mutation contribute to eosinophilic inflammatory bowel disease (70) whereas in Lewis rats experimental autoimmune encephalomyelitis (EAE) becomes aggravated (71).

In humans, transcripts for *GIMAP5* can be detected in peripheral blood T cells but not in B cells (62). Human *GIMAP5* was initially identified in 2001 as the Oar-2 clone from a Jurkat-derived cDNA library that could confer resistance to gamma-radiation and okadaic acid (OA)-induced apoptosis (72). In 2003, the protein was identified as Irod (inhibitor of radiation- and OA- induced death) (73). Overexpression of Oar-2 conferred protection in a CaMKII dependent manner in Jurkat cells (73). Regulation of sensitivity to OA may be species-specific, as no significant alterations in protein phosphatase activity was observed in *Gimap5^lyp/lyp^* rat T cells (28).

To better understand the functions of *Gimap5*, three groups generated mice with inactivating mutations in *Gimap5* (35–37). Two lines of *Gimap5*-deficient mice generated by the groups of H. Weiler and K. Hoebe exhibited comparable phenotype (35, 36). The Weiler laboratory generated *Gimap5^-/-^* mice by replacing the *Gimap5* exon 2 and a part of exon 3 with the neomycin cassette (36). The Hoebe group inactivated *Gimap5* through ENU mediated mutagenesis to generate the *Sphinx* (*Gimap5^sph/sph^*) line of mice (35). The latter carried a point mutation G38C that can abrogate the binding of GTP/GDP to a site that is conserved in RAS family of GTPases. Unlike in rats, where the defect caused by the *lyp* allele is restricted to T cells, absence of functional GIMAP5 leads to a paucity of peripheral T, B and NK cells in *Gimap5^-/-^* and in *Gimap5^sph/sph^* mice. However, positive and negative selection of T cells was not altered by the absence of GIMAP5 (35). In addition to T and B lymphopenia, both lines of mice show exhaustion of hematopoietic stem cells (HSC) and hepatic extramedullary hematopoiesis that is independent of T and B lymphocytes, as *Rag1^-/-^Gimap5^-/-^* mice also show the same phenotype (35, 38). In contrast to the above two mouse strains, *Gimap5^-/-^* mice developed by the Takahama group did not exhibit any T cell survival defects (37). Reasons for this discrepancy remains unclear. The *Sphinx* mice have also been reported to develop intestinal inflammation (35, 39).

### Mutation in the *Gimap5* Gene Disrupts Signaling Pathways in T Cells

**Figure 2:**
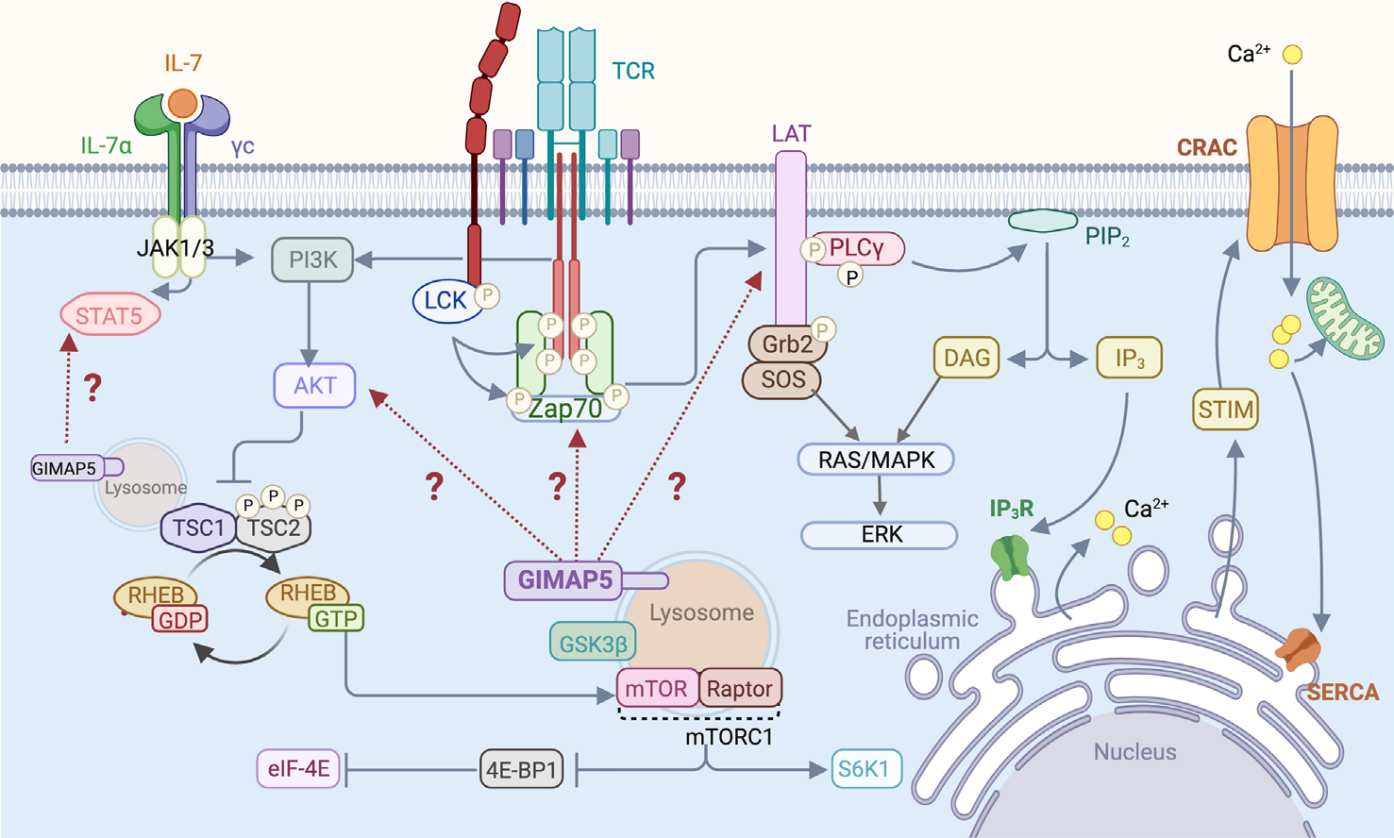
T cell signaling pathways that are influenced by GIMAP5. Following TCR stimulation by MHC:peptide complex or by Ab-mediated TCR cross-linking, LCK phosphorylates CD3 zeta chains and ZAP70, resulting in the phosphorylation of LAT that acts as a scaffold for downstream signaling molecules such as PLCγ. Activation of the PI3K/AKT signaling pathway downstream of TCR phosphorylates and inhibits the TSC1/2 complex, relieving repression of the mTORC1 kinase and leading to activation of downstream signaling events. IL-7 signaling stimulates STAT5 and also activates the PI3K/AKT pathway. GIMAP5 deficiency in rat and mouse T cells compromises proximal TCR signaling characterized by reduced Tyr phosphorylation of ZAP70 and LAT, but results in constitutive activation of AKT and mTORC1. GIMAP5 deficient T cells also display reduced IL-7-induced STAT5 phosphorylation. It is unclear how GIMAP5 impacts the TCR and IL-7R signaling pathways and regulates AKT activity (29, 34, 40–42).

Homeostatic survival of naive T cells requires two essential signals, one provided by the cytokine interleukin-7 (IL-7) and the other by MHC:self-peptide complexes that engage the TCR (74). Signals delivered *via* the IL-7 receptor and the TCR impact the classical pathway that maintains quiescence in most cell types involving LKB1 and AMPK (75, 76). AMPK, the energy sensor activated by an elevated AMP/ATP ratio, inhibits the mTORC1 complex by activating its suppressor, the TSC1/2 complex (77, 78). TCR engagement triggers the activation of LCK and ZAP70 that phosphorylate many substrates including the scaffolding protein LAT, resulting in the formation of multi-molecular signaling complex at the plasma membrane (79) (**Figure 2**). Activation of the PI3K/AKT signaling pathway downstream of TCR signaling phosphorylates TSC1/2 complex thereby releasing RHEB GTPase from suppression to activate the mTORC1 complex (80). mTORC1 promotes translation and protein synthesis by activating 70-kDa ribosomal S6 kinase (S6K1) and releasing the repressor protein 4E-BP1 from the translation initiation factor eIF-E4. Thus, in the absence of functional LKB1, AMPK or the TSC1/2 complex, T cell quiescence is lost (75–78). We observed that *Gimap5*-deficient T cells showed normal AMPK activation and mitochondrial respiration but displayed defects in IL-7 signaling, proximal TCR signaling manifested as reduced phosphorylation of ZAP70 and LAT, T cell calcium response and constitutive activation of AKT and the mTORC1 pathway (28–31) (**Figure 2**). Molecular mechanisms by which GIMAP5 impacts TCR and IL-7R signaling pathways remain to be elucidated.

### Rat GIMAP5 Regulates Cellular Calcium Homeostasis

**Figure 3:**
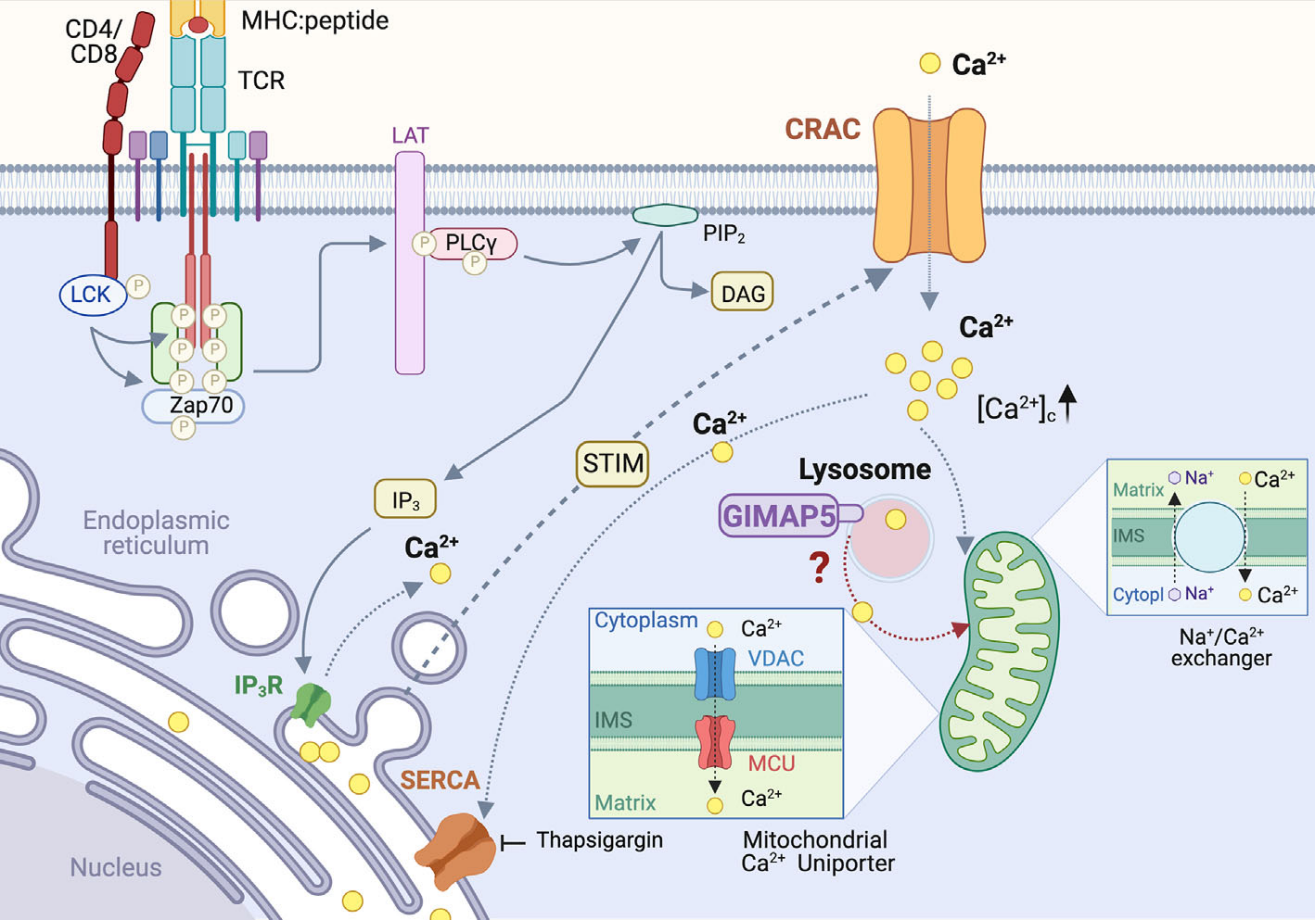
Regulation of Calcium homeostasis in rat T cells by GIMAP5. TCR-induced PLCγ activation leads to cleavage of plasma membrane-associated phosphatidylinositol 4,5 bisphosphate (PIP_2_) to generate inositol 1,4,5-triphosphate (IP_3_). IP_3_ binds to its receptor IP_3_R on the endoplasmic reticulum (ER) and triggers Ca^2+^ release from the ER store, resulting in a conformational change in the ER-localized STIM1 protein. This event relays a signal to open the Ca^2+^ release-activated Ca^2+^ channel (CRAC) on the plasma membrane, inducing the capacitative Ca^2+^ entry. The rising concentration of cytosolic calcium ([Ca^2+^]_c_) activates the Ca^2+^ uniporter on the mitochondrial membrane to uptake Ca^2+^, which is released later *via* the Na^+^/Ca^2+^ exchanger. In addition to ER, lysosomes also release a significant amount of Ca^2+^ following cell activation. Loss of GIMAP5 does not affect TCR- or thapsigargin- induced Ca^2+^ release from the ER stores but reduces Ca^2+^entry from extracellular milieu. GIMAP5 resides on lysosomes and the loss of GIMAP5 reduces lysosomal and mitochondrial Ca^2+^ content, presumably leading to feedback inhibition of the CRAC channels by cytosolic Ca^2+^. How GIMAP5 integrates TCR signaling to regulate lysosomal and mitochondrial Ca^2+^ to promote T cell survival and functions remains to be elucidated (28, 30, 31).

The TCR signalosome recruits and activates phospholipase Cγ (PLCγ), which hydrolyzes the membrane bound phosphatidylinositol 4,5 bisphosphate (PIP_2_) to generate inositol 1,4,5-triphosphate (IP_3_) and diacyl glycerol (DAG) (**Figure 2**). IP_3_ binds to its receptor IP_3_R on the endoplasmic reticulum (ER) and triggers Ca^2+^ release from the ER store, resulting in a conformational change in the ER-localized STIM1 protein (81, 82) (**Figure 3**). This event relays a signal to open the Ca^2+^ release-activated Ca^2+^ channel (CRAC) on the plasma membrane, inducing the capacitative Ca^2+^ entry from the extracellular milieu (83, 84). CRAC channels consisting of the ORAI proteins are the major store-operated channels in T lymphocytes (85, 86). TCR stimulation by antigen induces sustained Ca^2+^ influx *via* CRAC channels leading to T cell proliferation (87).

We observed that TCR-induced Ca^2+^ flux is reduced in T cells from BB-DP rats, which lack a functional GIMAP5 protein (30). The IP_3_-mediated Ca^2+^ release from the ER stores into the cytosol (88), can be mimicked by blocking the sarco/endoplasmic reticulum Ca^2+^ ATPases (SERCA) pump that refills the ER Ca^2+^ reserve, using thapsigargin (89) (**Figure 3**). We observed that GIMAP5 deficiency does not affect Ca^2+^ release from the ER in primary rat T cells (30). Similarly, overexpression of rGIMAP5 did not influence the emptying of the ER Ca^2+^ stores (31). However, Ca^2+^ influx from the extracellular milieu which occurs mainly *via* the CRAC channels, was reduced in *Gimap5-*deficient rat T cells (28, 30).

Following sustained Ca^2+^ entry *via* the CRAC channels, the rising concentration of cytosolic Ca^2+^ ([Ca^2+^]_c_) activates the Ca^2+^ uniporter on the mitochondrial membrane. This induces a slow, membrane potential-driven uptake of Ca^2+^, which is released later *via* the Na^+^/Ca^2+^ exchanger (90) (**Figure 3**). This process ensures that Ca^2+^ entering *via* the CRAC channel does not cause a feedback inhibition of the CRAC channel activity (91, 92). Thus, mitochondria act like a slow, non-saturable, non-linear buffer for intracellular Ca^2+^, as they sequester [Ca^2+^]_c_ during periods of rapid Ca^2+^ entry and sustain the [Ca^2+^]_c_ level by releasing it slowly even after the cessation of Ca^2+^ influx (91) (**Figure 3**). We observed that the reduced Ca^2+^ influx in *Gimap5*-deficient T cells is associated with the inability of their mitochondria to sequester Ca^2+^ ([Ca^2+^]_m_) following capacitative entry (28). This reduced mitochondrial Ca^2+^ was also observed following stimulation of the ryanodine receptors that are present on ER membrane (28) and are implicated in T cell functions (93–95). As a corollary, overexpression of rGIMAP5 in HEK293 cells resulted in increased Ca^2+^ accumulation within the mitochondria (28). As a consequence, Ca^2+^ influx from extracellular milieu is reduced in cells expressing GIMAP5, probably due to early saturation of the mitochondrial Ca^2+^ store (28).

**Figure 4:**
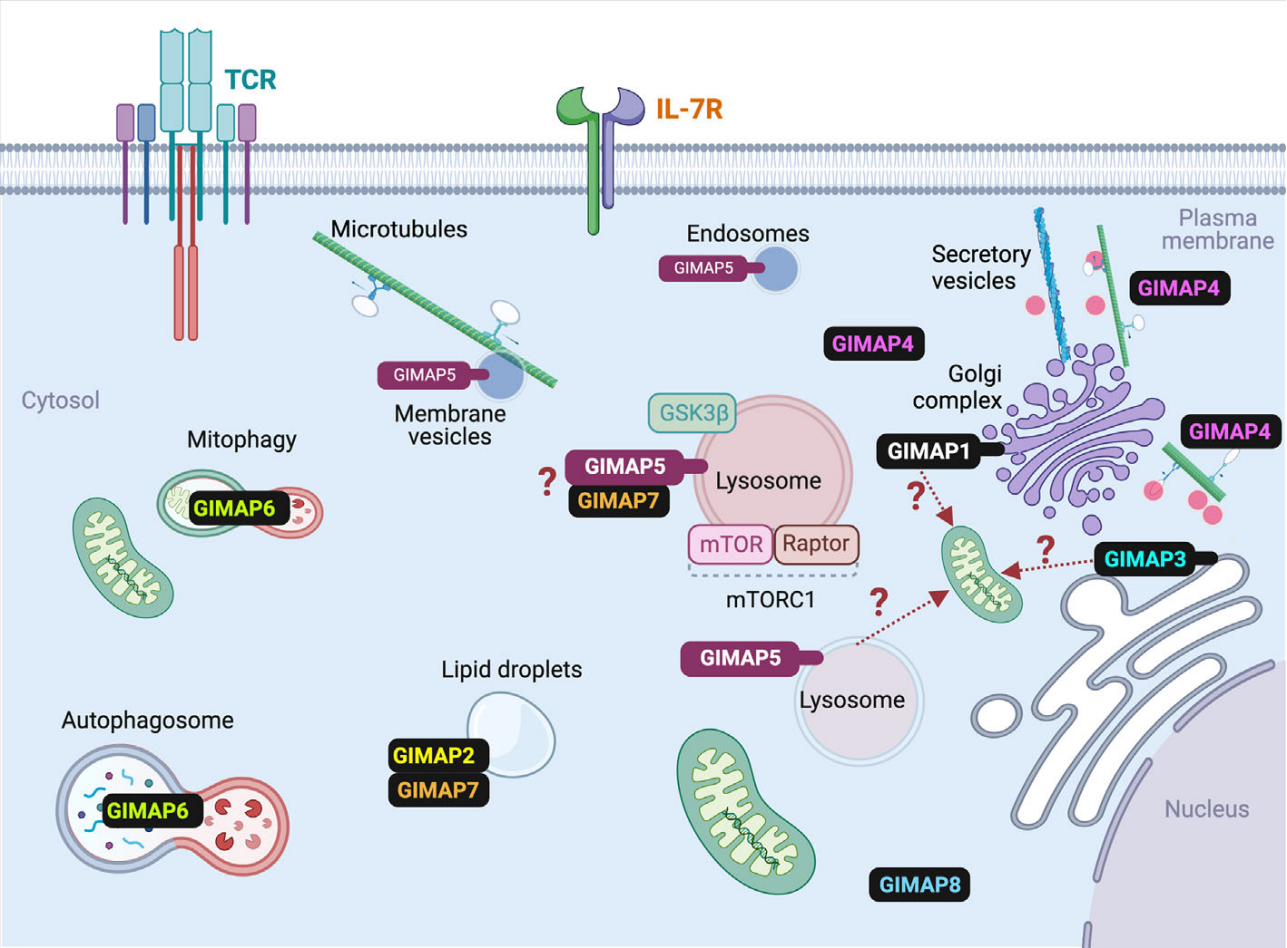
Subcellular localization of various GIMAP proteins in T cells. The known subcellular localization of mammalian GIMAP proteins in T lymphocytes is indicated. For the sake of simplicity, species-specific expression pattern of some GIMAP proteins are omitted. Details are given in the text and **Table 1**. Events that are predicted but require experimental confirmation are indicated by question marks. Ref- GIMAP5: (31–33); GIMAP1: (33); GIMAP2: (45); GIMAP3: (47); GIMAP4: (49); GIMAP6: (51); GIMAP7: (45); GIMAP8: (55).

Even though the ER is the major intracellular Ca^2+^ store, and mitochondria uptake Ca^2+^ to prevent feedback inhibition of the CRAC channels, a significant amount of Ca^2+^ can be released from the Golgi complex, lysosomes, nucleus and secretory granules (96). As GIMAP5 does not directly interact with mitochondria but is present on lysosomes and certain endocytic vesicles (31–33, 40), we postulated that GIMAP5 might regulate the Ca^2+^ content of lysosomes (**Figure 3**). Ca^2+^ was released from lysosomes by Gly-Phe β-naphthylamide (GPN) whose hydrolysis by cathepsin C results in osmotic lysis of the acidic compartment (97). We observed that GPN-mediated Ca^2+^ release was increased in T cells from *Gimap5^lyp/lyp^* rats, and the Ca^2+^ influx from the extracellular milieu was also higher than that of T cells from control rats (31). These observations suggest that the intracellular partitioning of Ca^2+^ in *Gimap5^lyp/lyp^* rat T cells is different from that of normal rat T cells. These observations were reflected in stable transfectants of full-length GIMAP5 in HEK293T cells, which displayed reduced GPN-induced Ca^2+^ release. Regulation of Ca^2+^ homeostasis was dependent on the full-length GIMAP5 protein as C-terminal or N-terminal deletion mutants were unable to do so. Further analyses of the truncated GIMAP5 proteins indicated that the GIMAP5 is anchored to lysosomal membranes and certain vesicles through the C-terminal anchor while the N-terminal regions interacted with the microtubules (31, 33) (**Figure 4**). Thus, the presence of GIMAP5 appears to decrease Ca^2+^ release from lysosomes. For the first time, our results suggest that lysosomal Ca^2+^ homeostasis regulates the survival of T cells and that this process requires GIMAP5. We also observed that that the lysosomal Ca^2+^ content was altered by signaling through the TCR in murine and human T cells (31). Whether and how GIMAP5 integrates TCR signaling to regulate lysosomal Ca^2+^ in order to promote T cell survival and functions remain to be elucidated (**Figure 3**).

### Signaling Pathways Affected in T Cells in the Absence of GIMAP5

Defective Ca^2+^ signaling in BB-DP rat T cells is evident within 30 minutes of TCR signaling induced by CD3 cross-linking (30). However, T cells from *Gimap5^Sph/Sph^* mice do not display any defect in Ca^2+^ influx induced by TCR signaling (40). Nonetheless, the TCR proximal signaling events showed similar impairments in *Gimap5* mutant T cells in both the species (29, 34, 40, 41) (**Figure 2**). Rat T cells were activated by cross-linking CD3/CD28, and T cells from OT-II TCR transgenic control and *Gimap5^sph/sph^* mice were activated using anti-CD3/CD28 antibodies or dendritic cells pulsed with the cognate OVA-peptide. *Gimap5* mutant rat and mouse T cells displayed reduced phosphorylation of LCK and the scaffold protein LAT following anti-CD3 stimulation (29). Notably, T cells from the *Gimap5* mutant mice and rats exhibited constitutive phosphorylation of ATK at the basal level (34). This constitutive phosphorylation of AKT was inhibited by PI3K inhibitors. While aberrant activation of the PI3K/AKT pathway results in malignant growth in most cell types (98), it causes cell death in non-transformed T lymphocytes, leading to immunodeficiency (99–101). Spontaneous activating mutations of PI3K that results in constitutively active PI3K/AKT pathway in T cells has been observed in a new class of patients with primary immunodeficiency (99–101). T cells from these patients do not proliferate in response to mitogenic signals. These published reports and our observation indicate that aberrant activation of the PI3K/AKT pathway in the absence of TCR engagement may result in T cell death. The constitutive phosphorylation of AKT can also explain the reduced phosphorylation of STAT5 that we have observed following IL-7 stimulation (29), as pAKT can suppress the phosphorylation of STAT5 (102).

Spontaneous AKT activation observed in *Gimap5* deficient T cells is reflected in the phosphorylation of downstream substrates including mTORC1 and FOXO1 (34). In fact, FOXO1 proteins are depleted progressively with age in *Gimap5^Sph/Sph^* mice (41). It is possible that the persistent phosphorylation of FOXO1 can lead to their degradation by proteasomes (103, 104). The Hoebe group recently showed that GIMAP5, which is localized on lysosomes and in certain vesicles, is required for+ the inactivation of GSK3β (40). GSK3β is a serine threonine kinase that is constitutively active in all cell types (105). Phosphorylation on Ser-9 and Ser-389 is required to inactivate GSK3β and promote T cell proliferation (106–108). Absence of GIMAP5 in T cells prevented CD3/CD28-induced inactivation of GSK3β as its inhibition by lithium chloride permitted the survival of T cells in *Gimap5^Sph/Sph^* mice (40). As GIMAP5 is present in certain vesicles, GIMAP5-mediated sequestration of GSK3β may play a role inhibiting its activity and promote cell proliferation (40).

### GIMAP5 in Hematopoietic Stem Cells

GIMAP5 is essential to maintain the hematopoietic stem cell (HSC) niche as HSC numbers diminish with age in mice carrying mutant GIMAP5 (38), although this is not the case in rats. The absolute numbers of HSCs as defined by Lin^-^IL-7R^-^cKit^+^Sca^+^ (LSK) cells, was comparable between wildtype and *Gimap5^-/-^* mice (38, 41). However, *Gimap*5-deficient HSCs have intrinsic defects in long-term engrafting capacity (38). The exhaustion of HSC in *Gimap5^-/-^* mice may be the consequence of their inability to stay in the quiescent state. In line with these observations, *Gimap5* expression is observed in murine HSC (38). Interaction of GIMAP5 with MCL1 and HSC70 appears to contribute to quiescence, however the underlying mechanisms are not yet known (38, 40). Simultaneous deletion of the two pro-apoptotic proteins, BAX and BAK, which rescues survival in the absence of MCL1, did not alter the survival of T cells in *Gimap5^Sph/Sph^* mice (40). It is possible that GSK3β, activated in the absence of GIMAP5 (40), might accelerate the degradation of MCL1 (109). These *in vivo* observations stand in contrast to over-expression studies *in vitro* where GIMAP5 was shown to interact with BCL-2 and BCL-xL (110). The requirement of GIMAP5 to maintain quiescence in HSCs is further supported by the observation that extramedullary hematopoiesis and reduced lifespan are still observed *Gimap5^-/-^Rag2^-/-^* mice (36).

Polymorphisms in human *GIMAP5* gene locus has been shown to be associated with autoimmune T1D and lupus in independent studies (111–114). Recently, mutations in GIMAP5 have been described in human primary immunodeficiency. The Hoebe group (40) described one patient who had mild lymphopenia with impaired T cell proliferation *in vitro* that was rescued by GSK3β inhibition. Lenardo’s group (42) identified 4 different family clusters with mutations in *GIMAP5* gene. Similar to what was reported in rats (31), GIMAP5 exists as 2 distinct isoforms in humans (42). GIMAP5 expression was observed in NK and T cells but not in B cells or monocytes. Nonetheless, frequency of T lymphocytes was reduced in all the patients studied while some of them also showed reduction in B lymphocytes and neutrophils. These patients exhibit splenomegaly and lymphadenopathy with abnormal liver and were susceptible to recurrent infections. T cells from the patients recapitulate the spontaneous mTORC1 activation observed in GIMAP5 mutant T cells from rats and mice (34). Similar to the *in vitro* findings (34), *Gimap5^Sph/Sph^* mice that received rapamycin, showed reduced activation of the mTORC1 pathway (42). Treatment of one of the patients with rapamycin for over 6 years diminished splenomegaly and lymphadenopathy, suggesting aberrant activation of the AKT/mTORC1 pathway *in vivo* in humans in the absence of functional GIMAP5. The abnormal activation of T cells in these patients is accompanied by replicative senescence in T cells as seen from reduced telomere length. These observations indicate that mutations in *Gimap5* profoundly compromises the survival of T cells. In rats only one of the two isoforms was shown to regulate calcium homeostasis (31). While the abundantly expressed GIMAP5v2 regulates lysosomal calcium, GIMAP5v1 that is expressed at a lower level did not. It appears that the GIMAP5v1 homolog is functional in mice and in humans but not in rats.

## GIMAP1

GIMAP1 was initially identified as *Imap38* that was induced in the spleen following *Plasmodium chaboudi* infection (115). However later studies carried out with anti-GIMAP1 antibody in infected tissues was unable to confirm these findings (116). GIMAP1, which is located in the Golgi complex (33), is expressed during all the stages of thymocyte development and in mature T, B cells and NK cells but minimally in macrophages (116). In humans, GIMAP1 is upregulated following Th1, but not Th2 differentiation *in vitro* (117). Constitutive and induced deletion of *Gimap1* in T cells compromised their survival in the periphery (43, 118). Following T cell activation *via* the TCR, *Gimap1^-/-^* CD4^+^ T cells upregulate markers of activation such as CD25 but fail to proliferate and expand (43). *In vitro*, *Gimap1^-/-^* CD4^+^ T cells showed accelerated loss of mitochondrial potential with a concomitant reduction in oxygen consumption. GIMAP1 is also essential for the survival and functioning of mature B cells (119). Transgenic expression of BCL-2 did not rescue the loss of *Gimap1^-/-^* B cells. Deletion of GIMAP1 in germinal center B cells prevented the generation of efficient T-dependent antibody responses. GIMAP1 expression is increased in diffuse large B-cell lymphoma (DLBCL) due to hypomethylation of the *GIMAP* locus (120). Additional studies are needed to understand the role of GIMAP1 in cell survival and leukemia.

## GIMAP2 and GIMAP7

Functional GIMAP2 is present in humans but is absent in mice and rats (3, 4). The functions of GIMAP2 have not been characterized. The C-terminal double hydrophobic domain, which is unique to GIMAP2, localizes it with the lipid droplets marker BIODIPY following overexpression in JURKAT cells (45). Structural studies of GTP-bound and unbound GIMAP2 indicate that the nucleotide binding domains of GIMAPs are related to those found in dynamin, chloroplast proteins Toc and septin-GTPases (45, 46). GTP binding induces the formation of dimers of GIMAP2 and presumably, of GIMAP5. It has been proposed that the membrane bound GIMAPs such as GIMAP5 and GIMAP2 may form scaffolds in the GTP associated forms (121). GTP hydrolysis may be initiated by dimerization-dependent mechanisms involving GIMAP7 and GIMAP4 that do not have a membrane anchor, to promote GTP catalysis (44, 63). If this hypothesis is supported by experimental evidence, it is possible that GTP-bound GIMAP5 scaffolds may inhibit apoptosis, whereas heterodimerization with GIMAP4 and consequent GTP hydrolysis may antagonize the pro-survival functions of GIMAP5 (44, 49). These studies may also help in identifying other associated proteins and delineating their functional contributions.

## GIMAP3

GIMAP3 is a pseudogene in humans and in rats (4). Mouse *Gimap3* is present in the ER (47) and influences the segregation of mitochondrial DNA in hematopoietic tissues (48). Mammalian mitochondrial DNA (mtDNA) is inherited from the mother. When all the cells in an organism carry the same haplotype of mtDNA it is referred to as homoplasmy (122, 123). In the case of mtDNA mutations, the cell carries two different haplotypes of mtDNA, referred to as heteroplasmy. Many of these mutations are associated with maternally inherited disorders in humans (122, 123). The two mitochondrial haplotypes are not inherited by daughter somatic cells in a stochastic manner (124). Additionally, mtDNA segregation in tissues and cell types are regulated by distinct mechanisms in an age-dependent manner (125). In inbred strains of *Mus musculus domesticus* mtDNA exists in two distinct haplotypes namely, BALB and NZB, which are transmitted in a random manner in the germline, but show segregation in the post-natal stage (126). For example, the BALB haplotype is enriched in hematopoietic cells while NZB is enriched in the liver and kidneys (127). While nuclear factors are implicated in tissue-specific segregation of mtDNA, the identity of proteins/RNA that could influence mtDNA segregation and the underlying mechanisms are not yet fully characterized (128, 129).

The group of Battersby showed that GIMAP3 is involved in regulating mtDNA segregation in hematopoietic cells (48). A mutation in the splice acceptor site of *Gimap3* in *Mus musculus castaneus* (CAST/Ei), results in *Gimap3* mRNA that codes for an additional 58 amino acids at the N-terminus (47). However, the longer N-terminus interferes with efficient translation, rendering CAST *Gimap3* a functional null mutant (47). Absence of functional *Gimap3* (CAST/Ei allele) resulted in equivalent representation of NZB mtDNA haplotype in hematopoietic tissues and in neutral tissues (where there is no haplotype selection), whereas mice expressing the wildtype *Giamp3* allele showed enrichment of BALB mtDNA haplotype in hematopoietic tissues (48). Analysis of segregation of BALB mtDNA in *Gimap5* heterozygous mice generated by the Weiler’s group (36) indicated that the abundance of GIMAP5 protein influenced that of GIMAP3 protein and was accompanied by differential segregation of BALB mtDNA in hematopoietic cells (47). In this context, it is interesting to note that Yano et al. (37), observed that T cell survival was significantly affected in *Gimap5/Gimap3* double knock out mice indicating as yet unknown interactions between these GIMAP members in hematopoietic cells.

## GIMAP4

*Gimap4* is expressed in developing T lymphocytes at the DN4 stage in response to pre-TCR signaling, is transiently downregulated in the DP stage and re-expressed in SP thymocytes, peripheral T cells as well as in B cells, NK cells and to a lesser extent in macrophages (49). The absence of GIMAP4 expression in RAG1-deficient thymocytes indicates that it is not expressed in DN 1-3 stages (49). Of the GIMAP proteins, interacting partners have been characterized in detail for GIMAP4 (49). GIMAP4 lacks a membrane anchor and is expressed in the cytosol (**Figure 4**). The C-terminal IQ domain, that is unique to the GIMAP4 (**Figure 1**) binds calmodulin. GIMAP4 harbours four PKC phosphorylation sites that are phosphorylated following T cell activation. Absence of GIMAP4 did not affect the generation or survival of T cells in the periphery. However, the frequency of apoptotic cells was increased in *Gimap4*-deficient T cells following exposure to gamma-irradiation, etoposide or dexamethasone, suggesting that GIMAP4 may be involved in promoting cell death following induction of apoptosis. In support of this notion, wildtype T cells undergoing apoptotic death display increased phosphorylation of GIMAP4 (49). The mitochondrial membrane potential and cytochrome c levels were comparable between the wildtype and *Gimap4*-deficient T cells indicating that GIMAP4 modulates apoptosis downstream of mitochondria. Furthermore, the apoptotic phenotype was inhibited by effector-caspase inhibitor in *Gimap4*-deficient T cells (49). Similar to other GIMAP proteins, GIMAP4 shows association with cytoskeletal elements and is implicated in membrane trafficking, movement of vesicles and cytokine secretion in T cells (31, 33, 47, 50, 130). Polymorphisms in *GIMAP4* was shown to be associated with asthma and allergy, although the underlying mechanisms remain to be elucidated (111).

## GIMAP6

Human GIMAP6 has been shown to be involved in autophagy (51). Mass spectrometry analysis identified GABARAPL2, the mammalian homolog of yeast ATG8, as the binding partner for GIMAP6. GIMAP6 is localized to the punctate structures along with GABARAPL2 and MAP1LC3B, an autophagosome marker. Knockdown of GIMAP6 in JURKAT T cells resulted in reduced levels of GABARAPL2, suggesting that GIMAP6 may regulate the expression of the latter (51). Knockdown of GIMAP6 in JURKAT cells also increased their sensitivity to apoptosis inducing agents (52). Similar to human GIMAP6, mouse GIMAP6 is also implicated in autophagy. CD2-Cre mediated deletion of *Gimap6* in mice caused in a significant reduction in T and B cell numbers in the periphery even though antigen-specific responses of *Gimap6^-/-^* T and B cells remained unaffected (53). The half-life of T cells lacking GIMAP6 was estimated to be around 4-5 days based on 4-hydroxytamoxifen mediated deletion in *Gimap6^fl/fl^ERT2Cre* mice. Recently, genetic loss of GIMAP6 protein was reported in humans, but with different degrees of clinical manifestations, presumably influenced by additional genetic and environmental factors, as one sibling exhibited lymphopenia while the other was asymptomatic even though both of them were homozygous for the mutant allele (54). Lymphocytes from these patients exhibited accelerated apoptosis, while maintaining normal activation, proliferation and cytokine secretion *in vitro*. Given the lymphocyte-specific expression of GIMAP proteins, it is possible that GIMAP6 may confer additional level of control over apoptotic and autophagic pathways in T cells.

## GIMAP8

In contrast to other GIMAP proteins, GIMAP8 possesses three GTPase domains and it is expressed in DN1, DN2 and mature thymocytes and T cells similar to GIMAP5 (23, 55). *Gimap8*-deficient mice show normal T cell development but show a reduction of recirculating B cells in the bone marrow (55). Nonetheless, the responses of B cells to a T-dependent antigen appear to be normal in these mice.

## GIMAP Genes in Leukemogenesis

The importance of GIMAP proteins in cell survival and their regulation during the ontogeny of different HSC-derived cell populations suggest that their deregulation may contribute to oncogenesis. The *GIMAP* locus has been implicated in T acute lymphoblastic leukemia (T-ALL) as a target of NOTCH signaling. During T cell development in the thymus, transition through DN1 to DN4 stages is accompanied by a progressive increase in NOTCH1 signaling (131). The DN3 to DN4 transition is a critical checkpoint that selects for productive rearrangement of the *TCRB* locus. Subsequent pre-TCR signaling induces NOTCH1 activation, which is required to expand the pool of cells that rearrange the *TCRA* locus, become CD4^+^CD8^+^ DP thymocytes and undergo positive and negative selection processes to generate SP CD4^+^ or CD8^+^ naïve T cells. A majority of thymocytes die due to the lack of survival signals in the absence of productively rearranged *TCR* genes (131, 132). Aberrant oncogenic signaling arising from faulty TCR rearrangement is often implicated in the pathogenesis of T-ALL. Aberrant NOTCH1 activation plays a key oncogenic role in T-ALL (133). In addition to NOTCH1, T-ALL development is associated with oncogenic activation of transcription factors such as TAL1, LYL1, LMO2, TLX1, TLX3 etc., which interfere with progression through T cell developmental stages (134–141).

GIMAP5 was identified as one of the NOTCH1 targets that contributed to the survival of leukemic T cell lines (142, 143). Many GIMAP genes are expressed in T-ALL cell lines (144, 145). The expression of *GIMAP5* occurs in many T cell leukemic cell lines and in anaplastic large cell lymphoma (ALCL) cell lines while the expression of GIMAP1, GIMAP2, GIMAP6 and GIMAP7 were down-regulated in ALCLs (44, 146). KMT2A/GIMAP8 rearrangements were detected in a patient with acute undifferentiated leukemia (147). *Gimap1* is upregulated in a murine leukemic cell line during p53-induced apoptosis (148). Most of the *Gimap* genes are expressed in HSC and/or in mature T cells but not in DN thymocytes in zebrafish (145). The *GIMAP* super-enhancer region was shown to be activated by the oncogenic transcription factor TAL1 and is repressed by E- proteins. Knocking out the TAL1 binding domain in JURKAT cells abrogated the expression of *GIMAP* genes. Ectopic expression of human *GIMAP5* and *GIMAP7* under the *Rag2* promoter did not induce leukemia but was capable of accelerating T-ALL induced by *MYC* in the zebrafish (145). As knockdown of *GIMAP* in T-ALL cells reduced their survival, it is possible that GIMAP proteins play an important role in maintaining the survival of transformed cells.

## Conclusions and Future Directions

GTPases with similarities to mammalian GIMAPs appear to have evolved independently in different species (56, 58, 59, 149–152). Proteins containing the AIG domain are present in angiosperms (flowering plants) and are induced by infections and stress (57, 61). *Gimap* genes are upregulated in certain invertebrates and zebrafish in response to infections (58, 59, 149, 151). Future studies aimed at understanding the functions of the related genes in the context of stress and infections in different species will further our knowledge on GIMAP proteins.

The data available to date overwhelmingly indicate that the GIMAP proteins have important and non-redundant functions in the survival of lymphocytes and in the maintenance of quiescence (GIMAP5) in HSCs. While some GIMAP proteins may interact with proteins involved in classical pathways of cell survival such as BCL2 family members and caspases, they also appear to promote survival by distinct mechanisms. GIMAP proteins are distinctly related to septins and share structural similarities with dynamins, Toc and other TRAnslation FACtor (TRAFAC) proteins (153). Structural analyses of GIMAP2 and GIAMP7 suggest that their GTPase activity is regulated by dimerization (44). GIMAP1, GIMAP2, GIMAP3 and GIMAP5 are membrane-associated while the rest of the GIMAP proteins do not have membrane anchor domains (**Figures 1**, **4**). Overexpressed GIMAP2 increases the formation of lipid droplets in JURKAT cells and can dimerize with GIMAP7 (44–46, 121). It is possible that the homo- and hetero- oligomers can form membrane scaffolds that recruit additional interacting proteins (121). Many of these interactions can be presumed to be dynamic and temporal, making it difficult to identify the interacting partners by classical methods (53). GIMAP5 and GIMAP4 have been observed to be associated with microtubules and actin (31, 50), implicating them in the transport of cellular cargo. Given the implication of GIMAP proteins in the survival of T lymphocytes, it is not surprising that GIMAPs have been associated with different types of leukemias. Unravelling the functions of GIMAP proteins will predicate a better understanding of their role in T cell survival and their contribution to the development of leukemias.

## Author Contributions

SR and M-AL designed the content and wrote the manuscript. SI, MC, and MN contributed to the discussions and specific sub-titles in the manuscript. All authors contributed to the article and approved the submitted version.

## Funding

This work was supported by SR’s NSERC Discovery grant RGPIN-2016-04349, MN’s FRQS post-doctoral fellowship #288811.

## Conflict of Interest

The authors declare that the research was conducted in the absence of any commercial or financial relationships that could be construed as a potential conflict of interest.

## Abbreviations

BB-DP, Biobreeding diabetes prone; CRAC, Ca^2+^ release activated Ca^2+^ channel; GIMAP, GTPase of the immune associated nucleotide binding protein; GPN, Gly-Phe β-naphthylamide; HSC, hematopoietic stem cell; MHC:p, MHC peptide complex; RTE, recent thymic emigrants; T1D, Type 1 diabetes.

## References

[1] Ramanathan S Poussier P. BB Rat Lyp Mutation and Type 1 Diabetes. Immunol Rev (2001) 184:161–71. 10.1034/j.1600-065x.2001.1840115.x 12086310

[2] Hornum L Romer J Markholst H. The Diabetes-Prone BB Rat Carries a Frameshift Mutation in Ian4, a Positional Candidate of Iddm1. Diabetes (2002) 51(6):1972–9. 10.2337/diabetes.51.6.1972 12031988

[3] MacMurray AJ Moralejo DH Kwitek AE Rutledge EA Van Yserloo B Gohlke P. Lymphopenia in the BB Rat Model of Type 1 Diabetes is Due to a Mutation in a Novel Immune-Associated Nucleotide (Ian)-Related Gene. Genome Res (2002) 12(7):1029–39. 10.1101/gr.412702 PMC186618 12097339

[4] Krucken J Schroetel RM Muller IU Saidani N Marinovski P Benten WP. Comparative Analysis of the Human Gimap Gene Cluster Encoding a Novel GTPase Family. Gene (2004) 341:291–304. 10.1016/j.gene.2004.07.005 15474311

[5] Colle E Guttmann RD Seemayer T. Spontaneous Diabetes Mellitus Syndrome in the Rat. I. Association With the Major Histocompatibility Complex. J Exp Med (1981) 154(4):1237–42. 10.1084/jem.154.4.1237 PMC2186484 7026724

[6] Jackson R Rassi N Crump T Haynes B Eisenbarth GS. The BB Diabetic Rat. Profound T-Cell Lymphocytopenia. Diabetes (1981) 30(10):887–9. 10.2337/diabetes.30.10.887 6974111

[7] Marliss EB Nakhooda AF Poussier P Sima AA. The Diabetic Syndrome of the ‘BB’ Wistar Rat: Possible Relevance to Type 1 (Insulin-Dependent) Diabetes in Man. Diabetologia (1982) 22(4):225–32. 10.1007/BF00281296 7047269

[8] Elder ME Maclaren NK. Identification of Profound Peripheral T Lymphocyte Immunodeficiencies in the Spontaneously Diabetic BB Rat. J Immunol (1983) 130(4):1723–31. 6220066

[9] Jacob HJ Pettersson A Wilson D Mao Y Lernmark A Lander ES. Genetic Dissection of Autoimmune Type I Diabetes in the BB Rat. Nat Genet (1992) 2(1):56–60. 10.1038/ng0992-56 1303251

[10] Poussier P Nakhooda AF Falk JA Lee C Marliss EB. Lymphopenia and Abnormal Lymphocyte Subsets in the “BB” Rat: Relationship to the Diabetic Syndrome. Endocrinology (1982) 110(5):1825–7. 10.1210/endo-110-5-1825 6978807

[11] Woda BA Padden C. BioBreeding/Worcester (BB/Wor) Rats are Deficient in the Generation of Functional Cytotoxic T Cells. J Immunol (1987) 139(5):1514–7. 3497974

[12] Prud’homme GJ Lapchak PH Parfrey NA Colle E Guttmann RD. Autoimmunity-Prone BB Rats Lack Functional Cytotoxic T Cells. Cell Immunol (1988) 114(1):198–208. 10.1016/0008-8749(88)90266-3 3131022

[13] Colle E Fuks A Poussier P Edouard P Guttmann RD. Polygenic Nature of Spontaneous Diabetes in the Rat. Permissive MHC Haplotype and Presence of the Lymphopenic Trait of the BB Rat are Not Sufficient to Produce Susceptibility. Diabetes (1992) 41(12):1617–23. 10.2337/diabetes.41.12.1617 1446803

[14] Sarkar P Crisa L McKeever U Bortell R Handler E Mordes JP. Loss of RT6 Message and Most Circulating T Cells After Thymectomy of Diabetes Prone BB Rats. Autoimmunity (1994) 18(1):15–22. 10.3109/08916939409014675 7999952

[15] Lau A Ramanathan S Poussier P. Excessive Production of Nitric Oxide by Macrophages From DP-BB Rats is Secondary to the T-Lymphopenic State of These Animals. Diabetes (1998) 47(2):197–205. 10.2337/diabetes.47.2.197 9519713

[16] Lee KU. Nitric Oxide Produced by Macrophages Mediates Suppression of ConA-Induced Proliferative Responses of Splenic Leukocytes in the Diabetes-Prone BB Rat. Diabetes (1994) 43(10):1218–20. 10.2337/diabetes.43.10.1218 7926291

[17] Ramanathan S Marandi L Poussier P. Evidence for the Extrathymic Origin of Intestinal TCRgammadelta(+) T Cells in Normal Rats and for an Impairment of This Differentiation Pathway in BB Rats. J Immunol (2002) 168(5):2182–7. 10.4049/jimmunol.168.5.2182 11859104

[18] Poussier P Ning T Murphy T Dabrowski D Ramanathan S. Impaired Post-Thymic Development of Regulatory CD4+25+ T Cells Contributes to Diabetes Pathogenesis in BB Rats. J Immunol (2005) 174(7):4081–9. 10.4049/jimmunol.174.7.4081 15778366

[19] Whalen BJ Greiner DL Mordes JP Rossini AA. Adoptive Transfer of Autoimmune Diabetes Mellitus to Athymic Rats: Synergy of CD4+ and CD8+ T Cells and Prevention by RT6+ T Cells. J Autoimmun (1994) 7(6):819–31. 10.1006/jaut.1994.1065 7888038

[20] Edouard P Hiserodt JC Plamondon C Poussier P. CD8+ T-cells are Required for Adoptive Transfer of the BB Rat Diabetic Syndrome. Diabetes (1993) 42(3):390–7. 10.2337/diabetes.42.3.390 8432409

[21] Zadeh HH Greiner DL Wu DY Tausche F Goldschneider I. Abnormalities in the Export and Fate of Recent Thymic Emigrants in Diabetes-Prone BB/W Rats. Autoimmunity (1996) 24(1):35–46. 10.3109/08916939608995355 8937686

[22] Groen H Klatter FA Brons NH Mesander G Nieuwenhuis P Kampinga J. Abnormal Thymocyte Subset Distribution and Differential Reduction of CD4+ and CD8+ T Cell Subsets During Peripheral Maturation in Diabetes-Prone BioBreeding Rats. J Immunol (1996) 156(3):1269–75. 8558007

[23] Dion C Carter C Hepburn L Coadwell WJ Morgan G Graham M. Expression of the Ian Family of Putative GTPases During T Cell Development and Description of an Ian With Three Sets of GTP/GDP-Binding Motifs. Int Immunol (2005) 17(9):1257–68. 10.1093/intimm/dxh302 16103028

[24] Plamondon C Kottis V Brideau C Metroz-Dayer MD Poussier P. Abnormal Thymocyte Maturation in Spontaneously Diabetic BB Rats Involves the Deletion of CD4-8+ Cells. J Immunol (1990) 144(3):923–8. 1688592

[25] Hernandez-Hoyos G Joseph S Miller NG Butcher GW. The Lymphopenia Mutation of the BB Rat Causes Inappropriate Apoptosis of Mature Thymocytes. Eur J Immunol (1999) 29(6):1832–41. 10.1002/(SICI)1521-4141(199906)29:06<1832::AID-IMMU1832>3.0.CO;2-F 10382745

[26] Lang JA Kominski D Bellgrau D Scheinman RI. Partial Activation Precedes Apoptotic Death in T Cells Harboring an IAN Gene Mutation. Eur J Immunol (2004) 34(9):2396–406. 10.1002/eji.200324751 15307172

[27] Ramanathan S Norwich K Poussier P. Antigen Activation Rescues Recent Thymic Emigrants From Programmed Cell Death in the BB Rat. J Immunol (1998) 160(12):5757–64. 9637485

[28] Chen XL Serrano D Mayhue M Wieden HJ Stankova J Boulay G. GTPase of the Immune-Associated Nucleotide-Binding Protein 5 (GIMAP5) Regulates Calcium Influx in T-lymphocytes by Promoting Mitochondrial Calcium Accumulation. Biochem J (2013) 449(2):353–64. 10.1042/BJ20120516 23098229

[29] Chen XL Serrano D Ghobadi F Mayhue M Hoebe K Ilangumaran S. TCR and IL-7 Signaling Are Altered in the Absence of Functional Gtpase of the Immune Associated Nucleotide Binding Protein 5 (GIMAP5). PloS One (2016) 11(3):e0151837. 10.1371/journal.pone.0151837 27023180 PMC4811415

[30] Ilangumaran S Forand-Boulerice M Bousquet SM Savard A Rocheleau P Chen XL. Loss of GIMAP5 (GTPase of Immunity-Associated Nucleotide Binding Protein 5) Impairs Calcium Signaling in Rat T Lymphocytes. Mol Immunol (2009) 46(6):1256–9. 10.1016/j.molimm.2008.09.031 19007993

[31] Serrano D Ghobadi F Boulay G Ilangumaran S Lavoie C Ramanathan S. GTPase of the Immune-Associated Nucleotide Protein 5 Regulates the Lysosomal Calcium Compartment in T Lymphocytes. Front Immunol (2017) 8:94. 10.3389/fimmu.2017.00094 28223986 PMC5293772

[32] Keita M Leblanc C Andrews D Ramanathan S. GIMAP5 Regulates Mitochondrial Integrity From a Distinct Subcellular Compartment. Biochem Biophys Res Commun (2007) 361(2):481–6. 10.1016/j.bbrc.2007.07.048 17655828

[33] Wong VW Saunders AE Hutchings A Pascall JC Carter C Bright NA. The Autoimmunity-Related GIMAP5 Gtpase is a Lysosome-Associated Protein. Self Nonself (2010) 1(3):259–68. 10.4161/self.1.3.12819 PMC3047789 21487483

[34] Chen XL Serrano D Mayhue M Hoebe K Ilangumaran S Ramanathan S. GIMAP5 Deficiency is Associated With Increased AKT Activity in T Lymphocytes. PloS One (2015) 10(10):e0139019. 10.1371/journal.pone.0139019 26440416 PMC4595448

[35] Barnes MJ Aksoylar H Krebs P Bourdeau T Arnold CN Xia Y. Loss of T Cell and B Cell Quiescence Precedes the Onset of Microbial Flora-Dependent Wasting Disease and Intestinal Inflammation in Gimap5-Deficient Mice. J Immunol (2010) 184(7):3743–54. 10.4049/jimmunol.0903164 PMC4395861 20190135

[36] Schulteis RD Chu H Dai X Chen Y Edwards B Haribhai D. Impaired Survival of Peripheral T Cells, Disrupted NK/NKT Cell Development, and Liver Failure in Mice Lacking Gimap5. Blood (2008) 112(13):4905–14. 10.1182/blood-2008-03-146555 PMC2597598 18796632

[37] Yano K Carter C Yoshida N Abe T Yamada A Nitta T. Gimap3 and Gimap5 Cooperate to Maintain T-Cell Numbers in the Mouse. Eur J Immunol (2014) 44(2):561–72. 10.1002/eji.201343750 24510501

[38] Chen Y Yu M Dai X Zogg M Wen R Weiler H. Critical Role for Gimap5 in the Survival of Mouse Hematopoietic Stem and Progenitor Cells. J Exp Med (2011) 208(5):923–35. 10.1084/jem.20101192 PMC3092340 21502331

[39] Endale M Aksoylar HI Hoebe K. Central Role of Gimap5 in Maintaining Peripheral Tolerance and T Cell Homeostasis in the Gut. Mediators Inflamm (2015) 2015:436017. 10.1155/2015/436017 25944983 PMC4405212

[40] Patterson AR Endale M Lampe K Aksoylar HI Flagg A Woodgett JR. Gimap5-Dependent Inactivation of GSK3beta is Required for CD4(+) T Cell Homeostasis and Prevention of Immune Pathology. Nat Commun (2018) 9(1):430. 10.1038/s41467-018-02897-7 29382851 PMC5789891

[41] Aksoylar HI Lampe K Barnes MJ Plas DR Hoebe K. Loss of Immunological Tolerance in Gimap5-Deficient Mice is Associated With Loss of Foxo in CD4+ T Cells. J Immunol (2012) 188(1):146–54. 10.4049/jimmunol.1101206 PMC3258489 22106000

[42] Park AY Leney-Greene M Lynberg M Xu X Zheng L Zhang Y. Human Immunodeficiency Reveals GIMAP5 as Lymphocyte-Specific Regulator of Senescence. bioRxiv (2021) 2021.02.22.432146. 10.1101/2021.02.22.432146

[43] Datta P Webb LM Avdo I Pascall J Butcher GW. Survival of Mature T Cells in the Periphery is Intrinsically Dependent on GIMAP1 in Mice. Eur J Immunol (2017) 47(1):84–93. 10.1002/eji.201646599 27792288 PMC5244661

[44] Schwefel D Arasu BS Marino SF Lamprecht B Kochert K Rosenbaum E. Structural Insights Into the Mechanism of GTPase Activation in the GIMAP Family. Structure (2013) 21(4):550–9. 10.1016/j.str.2013.01.014 23454188

[45] Schwefel D Frohlich C Eichhorst J Wiesner B Behlke J Aravind L. Structural Basis of Oligomerization in Septin-Like GTPase of Immunity-Associated Protein 2 (GIMAP2). Proc Natl Acad Sci U S A (2010) 107(47):20299–304. 10.1073/pnas.1010322107 PMC2996681 21059949

[46] Schwefel D Frohlich C Daumke O. Purification, Crystallization and Preliminary X-ray Analysis of Human GIMAP2. Acta Crystallogr Sect F Struct Biol Cryst Commun (2010) 66(Pt 6):725–9. 10.1107/S174430911001537X PMC2882781 20516611

[47] Jokinen R Lahtinen T Marttinen P Myohanen M Ruotsalainen P Yeung N. Quantitative Changes in Gimap3 and Gimap5 Expression Modify Mitochondrial DNA Segregation in Mice. Genetics (2015) 200(1):221–35. 10.1534/genetics.115.175596 PMC4423365 25808953

[48] Jokinen R Marttinen P Sandell HK Manninen T Teerenhovi H Wai T. Gimap3 Regulates Tissue-Specific Mitochondrial DNA Segregation. PloS Genet (2010) 6(10):e1001161. 10.1371/journal.pgen.1001161 20976251 PMC2954831

[49] Schnell S Demolliere C van den Berk P Jacobs H. Gimap4 Accelerates T-cell Death. Blood (2006) 108(2):591–9. 10.1182/blood-2005-11-4616 16569770

[50] Heinonen MT Kanduri K Lahdesmaki HJ Lahesmaa R Henttinen TA. Tubulin- and Actin-Associating GIMAP4 is Required for IFN-gamma Secretion During Th Cell Differentiation. Immunol Cell Biol (2015) 93(2):158–66. 10.1038/icb.2014.86 PMC4355353 25287446

[51] Pascall JC Rotondo S Mukadam AS Oxley D Webster J Walker SA. The Immune System GTPase GIMAP6 Interacts With the Atg8 Homologue GABARAPL2 and is Recruited to Autophagosomes. PloS One (2013) 8(10):e77782. 10.1371/journal.pone.0077782 24204963 PMC3804274

[52] Ho CH Tsai SF. Functional and Biochemical Characterization of a T Cell-Associated Anti-Apoptotic Protein, GIMAP6. J Biol Chem (2017) 292(22):9305–19. 10.1074/jbc.M116.768689 PMC5454111 28381553

[53] Pascall JC Webb LMC Eskelinen EL Innocentin S Attaf-Bouabdallah N Butcher GW. GIMAP6 is Required for T Cell Maintenance and Efficient Autophagy in Mice. PloS One (2018) 13(5):e0196504. 10.1371/journal.pone.0196504 29718959 PMC5931655

[54] Shadur B Asherie N Kfir-Erenfeld S Dubnikov T NaserEddin A Schejter YD. A Human Case of GIMAP6 Deficiency: A Novel Primary Immune Deficiency. Eur J Hum Genet (2020) 29(4):657–62. 10.1038/s41431-020-00773-x PMC7739214 33328581

[55] Webb LM Pascall JC Hepburn L Carter C Turner M Butcher GW. Generation and Characterisation of Mice Deficient in the Multi-GTPase Domain Containing Protein, GIMAP8. PloS One (2014) 9(10):e110294. 10.1371/journal.pone.0110294 25329815 PMC4201521

[56] Weinstein DJ Allen SE Lau MCY Erasmus M Asalone KC Walters-Conte K. The Genome of a Subterrestrial Nematode Reveals Adaptations to Heat. Nat Commun (2019) 10(1):5268. 10.1038/s41467-019-13245-8 31754114 PMC6872716

[57] Wang Z Li X. IAN/GIMAPs are Conserved and Novel Regulators in Vertebrates and Angiosperm Plants. Plant Signal Behav (2009) 4(3):165–7. 10.4161/psb.4.3.7722 PMC2652520 19721741

[58] Weiss Y Foret S Hayward DC Ainsworth T King R Ball EE. The Acute Transcriptional Response of the Coral Acropora Millepora to Immune Challenge: Expression of GiMAP/IAN Genes Links the Innate Immune Responses of Corals With Those of Mammals and Plants. BMC Genomics (2013) 14:400. 10.1186/1471-2164-14-400 23768317 PMC3723955

[59] Lu L Loker ES Zhang SM Buddenborg SK Bu L. Genome-Wide Discovery, and Computational and Transcriptional Characterization of an AIG Gene Family in the Freshwater Snail Biomphalaria Glabrata, a Vector for Schistosoma Mansoni. BMC Genomics (2020) 21(1):190. 10.1186/s12864-020-6534-z 32122294 PMC7053062

[60] Liu C Wang T Zhang W Li X. Computational Identification and Analysis of Immune-Associated Nucleotide Gene Family in Arabidopsis Thaliana. J Plant Physiol (2008) 165(7):777–87. 10.1016/j.jplph.2007.06.002 17723251

[61] Reuber TL Ausubel FM. Isolation of Arabidopsis Genes That Differentiate Between Resistance Responses Mediated by the RPS2 and RPM1 Disease Resistance Genes. Plant Cell (1996) 8(2):241–9. 10.1105/tpc.8.2.241 PMC161094 8742710

[62] Zenz T Roessner A Thomas A Frohling S Dohner H Calabretta B. hIan5: The Human Ortholog to the Rat Ian4/Iddm1/lyp is a New Member of the Ian Family That is Overexpressed in B-Cell Lymphoid Malignancies. Genes Immun (2004) 5(2):109–16. 10.1038/sj.gene.6364044 14724691

[63] Cambot M Aresta S Kahn-Perles B de Gunzburg J Romeo PH. Human Immune Associated Nucleotide 1: A Member of a New Guanosine Triphosphatase Family Expressed in Resting T and B Cells. Blood (2002) 99(9):3293–301. 10.1182/blood.V99.9.3293 11964296

[64] Filen S Lahesmaa R. Gimap Proteins in T-Lymphocytes. J Signal Transduct (2010) 2010:268589. 10.1155/2010/268589 21637352 PMC3100574

[65] Yui MA Rothenberg EV. Developmental Gene Networks: A Triathlon on the Course to T Cell Identity. Nat Rev Immunol (2014) 14(8):529–45. 10.1038/nri3702 PMC4153685 25060579

[66] Ashton-Rickardt PG Van Kaer L Schumacher TN Ploegh HL Tonegawa S. Peptide Contributes to the Specificity of Positive Selection of CD8+ T Cells in the Thymus. Cell (1993) 73(5):1041–9. 10.1016/0092-8674(93)90281-t 8500174

[67] Klein L Hinterberger M Wirnsberger G Kyewski B. Antigen Presentation in the Thymus for Positive Selection and Central Tolerance Induction. Nat Rev Immunol (2009) 9(12):833–44. 10.1038/nri2669 19935803

[68] Kirberg J Berns A von Boehmer H. Peripheral T Cell Survival Requires Continual Ligation of the T Cell Receptor to Major Histocompatibility Complex-Encoded Molecules. J Exp Med (1997) 186(8):1269–75. 10.1084/jem.186.8.1269 PMC2199081 9334366

[69] Modigliani Y Coutinho G Burlen-Defranoux O Coutinho A Bandeira A. Differential Contribution of Thymic Outputs and Peripheral Expansion in the Development of Peripheral T Cell Pools. Eur J Immunol (1994) 24(5):1223–7. 10.1002/eji.1830240533 8181533

[70] Cousins L Graham M Tooze R Carter C Miller JR Powrie FM. Eosinophilic Bowel Disease Controlled by the BB Rat-Derived Lymphopenia/Gimap5 Gene. Gastroenterology (2006) 131(5):1475–85. 10.1053/j.gastro.2006.09.023 17064701

[71] Fischer HJ Witte AK Walter L Grone HJ van den Brandt J Reichardt HM. Distinct Roles of T-Cell Lymphopenia and the Microbial Flora for Gastrointestinal and CNS Autoimmunity. FASEB J (2016) 30(5):1724–32. 10.1096/fj.15-277384 26740263

[72] Sandal T Ahlgren R Lillehaug J Doskeland SO. Establishment of Okadaic Acid Resistant Cell Clones Using a cDNA Expression Library. Cell Death Differ (2001) 8(7):754–66. 10.1038/sj.cdd.4400873 11464220

[73] Sandal T Aumo L Hedin L Gjertsen BT Doskeland SO. Irod/Ian5: An Inhibitor of Gamma-Radiation- and Okadaic Acid-Induced Apoptosis. Mol Biol Cell (2003) 14(8):3292–304. 10.1091/mbc.E02-10-0700 PMC181568 12925764

[74] Takada K Jameson SC. Self-Class I MHC Molecules Support Survival of Naive CD8 T Cells, But Depress Their Functional Sensitivity Through Regulation of CD8 Expression Levels. J Exp Med (2009) 206(10):2253–69. 10.1084/jem.20082553 PMC2757867 19752186

[75] MacIver NJ Blagih J Saucillo DC Tonelli L Griss T Rathmell JC. The Liver Kinase B1 is a Central Regulator of T Cell Development, Activation, and Metabolism. J Immunol (2011) 187(8):4187–98. 10.4049/jimmunol.1100367 PMC3206094 21930968

[76] Blagih J Krawczyk CM Jones RG. LKB1 and AMPK: Central Regulators of Lymphocyte Metabolism and Function. Immunol Rev (2012) 249(1):59–71. 10.1111/j.1600-065X.2012.01157.x 22889215

[77] Wu Q Liu Y Chen C Ikenoue T Qiao Y Li CS. The Tuberous Sclerosis Complex-Mammalian Target of Rapamycin Pathway Maintains the Quiescence and Survival of Naive T Cells. J Immunol (2011) 187(3):1106–12. 10.4049/jimmunol.1003968 PMC3151493 21709159

[78] Yang K Neale G Green DR He W Chi H. The Tumor Suppressor Tsc1 Enforces Quiescence of Naive T Cells to Promote Immune Homeostasis and Function. Nat Immunol (2011) 12(9):888–97. 10.1038/ni.2068 PMC3158818 21765414

[79] Malissen B Gregoire C Malissen M Roncagalli R. Integrative Biology of T Cell Activation. Nat Immunol (2014) 15(9):790–7. 10.1038/ni.2959 25137453

[80] Powell JD Pollizzi KN Heikamp EB Horton MR. Regulation of Immune Responses by mTOR. Annu Rev Immunol (2012) 30:39–68. 10.1146/annurev-immunol-020711-075024 22136167 PMC3616892

[81] Liou J Kim ML Heo WD Jones JT Myers JW Ferrell JE Jr.. STIM is a Ca2+ Sensor Essential for Ca2+-Store-Depletion-Triggered Ca2+ Influx. Curr Biol (2005) 15(13):1235–41. 10.1016/j.cub.2005.05.055 PMC3186072 16005298

[82] Roos J DiGregorio PJ Yeromin AV Ohlsen K Lioudyno M Zhang S. STIM1, an Essential and Conserved Component of Store-Operated Ca2+ Channel Function. J Cell Biol (2005) 169(3):435–45. 10.1083/jcb.200502019 PMC2171946 15866891

[83] Berridge MJ. Inositol Trisphosphate and Calcium Signalling. Nature (1993) 361(6410):315–25. 10.1038/361315a0 8381210

[84] Hogan PG Lewis RS Rao A. Molecular Basis of Calcium Signaling in Lymphocytes: STIM and ORAI. Annu Rev Immunol (2010) 28:491–533. 10.1146/annurev.immunol.021908.132550 20307213 PMC2861828

[85] Feske S. Calcium Signalling in Lymphocyte Activation and Disease. Nat Rev Immunol (2007) 7(9):690–702. 10.1038/nri2152 17703229

[86] Feske S Gwack Y Prakriya M Srikanth S Puppel SH Tanasa B. A Mutation in Orai1 Causes Immune Deficiency by Abrogating CRAC Channel Function. Nature (2006) 441(7090):179–85. 10.1038/nature04702 16582901

[87] Valitutti S Dessing M Aktories K Gallati H Lanzavecchia A. Sustained Signaling Leading to T Cell Activation Results From Prolonged T Cell Receptor Occupancy. Role of T Cell Actin Cytoskeleton. J Exp Med (1995) 181(2):577–84. 10.1084/jem.181.2.577 PMC2191861 7836913

[88] Lewis RS. Calcium Signaling Mechanisms in T Lymphocytes. Annu Rev Immunol (2001) 19:497–521. 10.1146/annurev.immunol.19.1.497 11244045

[89] Thastrup O Cullen PJ Drobak BK Hanley MR Dawson AP. Thapsigargin, a Tumor Promoter, Discharges Intracellular Ca2+ Stores by Specific Inhibition of the Endoplasmic Reticulum Ca2(+)-Atpase. Proc Natl Acad Sci U S A (1990) 87(7):2466–70. 10.1073/pnas.87.7.2466 PMC53710 2138778

[90] Pfeiffer DR Gunter TE Eliseev R Broekemeier KM Gunter KK. Release of Ca2+ From Mitochondria Via the Saturable Mechanisms and the Permeability Transition. IUBMB Life (2001) 52(3-5):205–12. 10.1080/15216540152846019 11798034

[91] Hoth M Fanger CM Lewis RS. Mitochondrial Regulation of Store-Operated Calcium Signaling in T Lymphocytes. J Cell Biol (1997) 137(3):633–48. 10.1083/jcb.137.3.633 PMC2139882 9151670

[92] Hajnoczky G Csordas G Yi M. Old Players in a New Role: Mitochondria-Associated Membranes, VDAC, and Ryanodine Receptors as Contributors to Calcium Signal Propagation From Endoplasmic Reticulum to the Mitochondria. Cell Calcium (2002) 32(5-6):363–77. 10.1016/s0143416002001872 12543096

[93] Conrad DM Hanniman EA Watson CL Mader JS Hoskin DW. Ryanodine Receptor Signaling is Required for Anti-CD3-Induced T Cell Proliferation, Interleukin-2 Synthesis, and Interleukin-2 Receptor Signaling. J Cell Biochem (2004) 92(2):387–99. 10.1002/jcb.20064 15108363

[94] Hakamata Y Nishimura S Nakai J Nakashima Y Kita T Imoto K. Involvement of the Brain Type of Ryanodine Receptor in T-Cell Proliferation. FEBS Lett (1994) 352(2):206–10. 10.1016/0014-5793(94)00955-4 7523185

[95] Hosoi E Nishizaki C Gallagher KL Wyre HW Matsuo Y Sei Y. Expression of the Ryanodine Receptor Isoforms in Immune Cells. J Immunol (2001) 167(9):4887–94. 10.4049/jimmunol.167.9.4887 11673493

[96] Rizzuto R Pozzan T. Microdomains of Intracellular Ca2+: Molecular Determinants and Functional Consequences. Physiol Rev (2006) 86(1):369–408. 10.1152/physrev.00004.2005 16371601

[97] Haller T Volkl H Deetjen P Dietl P. The Lysosomal Ca2+ Pool in MDCK Cells can be Released by Ins(1,4,5)P3-Dependent Hormones or Thapsigargin But Does Not Activate Store-Operated Ca2+ Entry. Biochem J (1996) 319( Pt 3):909–12. 10.1042/bj3190909 PMC1217874 8920998

[98] Curran E Smith SM. Phosphoinositide 3-Kinase Inhibitors in Lymphoma. Curr Opin Oncol (2014) 26(5):469–75. 10.1097/CCO.0000000000000113 PMC4441952 25024054

[99] Thauvin-Robinet C Auclair M Duplomb L Caron-Debarle M Avila M St-Onge J. PIK3R1 Mutations Cause Syndromic Insulin Resistance With Lipoatrophy. Am J Hum Genet (2013) 93(1):141–9. 10.1016/j.ajhg.2013.05.019 PMC3710759 23810378

[100] Deau MC Heurtier L Frange P Suarez F Bole-Feysot C Nitschke P. A Human Immunodeficiency Caused by Mutations in the PIK3R1 Gene. J Clin Invest (2014) 124(9):3923–8. 10.1172/JCI75746 PMC4153704 25133428

[101] Lucas CL Kuehn HS Zhao F Niemela JE Deenick EK Palendira U. Dominant-Activating Germline Mutations in the Gene Encoding the PI(3)K Catalytic Subunit p110delta Result in T Cell Senescence and Human Immunodeficiency. Nat Immunol (2014) 15(1):88–97. 10.1038/ni.2771 24165795 PMC4209962

[102] Hand TW Cui W Jung YW Sefik E Joshi NS Chandele A. Differential Effects of STAT5 and PI3K/AKT Signaling on Effector and Memory CD8 T-Cell Survival. Proc Natl Acad Sci U S A (2010) 107(38):16601–6. 10.1073/pnas.1003457107 PMC2944719 20823247

[103] Aoki M Jiang H Vogt PK. Proteasomal Degradation of the FoxO1 Transcriptional Regulator in Cells Transformed by the P3k and Akt Oncoproteins. Proc Natl Acad Sci U S A (2004) 101(37):13613–7. 10.1073/pnas.0405454101 PMC518802 15342912

[104] Huang H Tindall DJ. Regulation of FOXO Protein Stability Via Ubiquitination and Proteasome Degradation. Biochim Biophys Acta (2011) 1813(11):1961–4. 10.1016/j.bbamcr.2011.01.007 PMC3110514 21238503

[105] Woodgett JR. Molecular Cloning and Expression of Glycogen Synthase Kinase-3/factor A. EMBO J (1990) 9(8):2431–8. 10.1002/j.1460-2075.1990.tb07419.x PMC552268 2164470

[106] Stambolic V Woodgett JR. Mitogen Inactivation of Glycogen Synthase Kinase-3 Beta in Intact Cells Via Serine 9 Phosphorylation. Biochem J (1994) 303( Pt 3):701–4. 10.1042/bj3030701 PMC1137602 7980435

[107] Thornton TM Pedraza-Alva G Deng B Wood CD Aronshtam A Clements JL. Phosphorylation by P38 MAPK as an Alternative Pathway for GSK3beta Inactivation. Science (2008) 320(5876):667–70. 10.1126/science.1156037 PMC2597039 18451303

[108] Thornton TM Delgado P Chen L Salas B Krementsov D Fernandez M. Inactivation of Nuclear GSK3beta by Ser(389) Phosphorylation Promotes Lymphocyte Fitness During DNA Double-Strand Break Response. Nat Commun (2016) 7:10553. 10.1038/ncomms10553 26822034 PMC4740185

[109] Maurer U Charvet C Wagman AS Dejardin E Green DR. Glycogen Synthase Kinase-3 Regulates Mitochondrial Outer Membrane Permeabilization and Apoptosis by Destabilization of MCL-1. Mol Cell (2006) 21(6):749–60. 10.1016/j.molcel.2006.02.009 16543145

[110] Nitta T Nasreen M Seike T Goji A Ohigashi I Miyazaki T. IAN Family Critically Regulates Survival and Development of T Lymphocytes. PloS Biol (2006) 4(4):e103. 10.1371/journal.pbio.0040103 16509771 PMC1393758

[111] Heinonen MT Laine AP Soderhall C Gruzieva O Rautio S Melen E. GIMAP GTPase Family Genes: Potential Modifiers in Autoimmune Diabetes, Asthma, and Allergy. J Immunol (2015) 194(12):5885–94. 10.4049/jimmunol.1500016 PMC4456634 25964488

[112] Hellquist A Zucchelli M Kivinen K Saarialho-Kere U Koskenmies S Widen E. The Human GIMAP5 Gene has a Common Polyadenylation Polymorphism Increasing Risk to Systemic Lupus Erythematosus. J Med Genet (2007) 44(5):314–21. 10.1136/jmg.2006.046185 PMC2597989 17220214

[113] Lim MK Sheen DH Kim SA Won SK Lee SS Chae SC. IAN5 Polymorphisms are Associated With Systemic Lupus Erythematosus. Lupus (2009) 18(12):1045–52. 10.1177/0961203309106830 19762377

[114] Shin JH Janer M McNeney B Blay S Deutsch K Sanjeevi CB. IA-2 Autoantibodies in Incident Type I Diabetes Patients are Associated With a Polyadenylation Signal Polymorphism in GIMAP5. Genes Immun (2007) 8(6):503–12. 10.1038/sj.gene.6364413 17641683

[115] Krucken J Schmitt-Wrede HP Markmann-Mulisch U Wunderlich F. Novel Gene Expressed in Spleen Cells Mediating Acquired Testosterone-Resistant Immunity to Plasmodium Chabaudi Malaria. Biochem Biophys Res Commun (1997) 230(1):167–70. 10.1006/bbrc.1996.5876 9020038

[116] Saunders A Lamb T Pascall J Hutchings A Dion C Carter C. Expression of GIMAP1, a GTPase of the Immunity-Associated Protein Family, is Not Up-Regulated in Malaria. Malar J (2009) 8:53. 10.1186/1475-2875-8-53 19338674 PMC2669093

[117] Filen JJ Filen S Moulder R Tuomela S Ahlfors H West A. Quantitative Proteomics Reveals GIMAP Family Proteins 1 and 4 to be Differentially Regulated During Human T Helper Cell Differentiation. Mol Cell Proteomics (2009) 8(1):32–44. 10.1074/mcp.M800139-MCP200 18701445 PMC2621005

[118] Saunders A Webb LM Janas ML Hutchings A Pascall J Carter C. Putative GTPase GIMAP1 is Critical for the Development of Mature B and T Lymphocytes. Blood (2010) 115(16):3249–57. 10.1182/blood-2009-08-237586 20194894

[119] Webb LM Datta P Bell SE Kitamura D Turner M Butcher GW. GIMAP1 Is Essential for the Survival of Naive and Activated B Cells In Vivo. J Immunol (2016) 196(1):207–16. 10.4049/jimmunol.1501582 PMC4685748 26621859

[120] Chambwe N Kormaksson M Geng H De S Michor F Johnson NA. Variability in DNA Methylation Defines Novel Epigenetic Subgroups of DLBCL Associated With Different Clinical Outcomes. Blood (2014) 123(11):1699–708. 10.1182/blood-2013-07-509885 PMC3954051 24385541

[121] Schwefel D Daumke O. GTP-Dependent Scaffold Formation in the GTPase of Immunity Associated Protein Family. Small GTPases (2011) 2(1):27–30. 10.4161/sgtp.2.1.14938 21686278 PMC3116615

[122] Wai T Teoli D Shoubridge EA. The Mitochondrial DNA Genetic Bottleneck Results From Replication of a Subpopulation of Genomes. Nat Genet (2008) 40(12):1484–8. 10.1038/ng.258 19029901

[123] Taylor RW Turnbull DM. Mitochondrial DNA Mutations in Human Disease. Nat Rev Genet (2005) 6(5):389–402. 10.1038/nrg1606 15861210 PMC1762815

[124] Chinnery PF Thorburn DR Samuels DC White SL Dahl HM Turnbull DM. The Inheritance of Mitochondrial DNA Heteroplasmy: Random Drift, Selection or Both? Trends Genet (2000) 16(11):500–5. 10.1016/s0168-9525(00)02120-x 11074292

[125] Jokinen R Junnila H Battersby BJ. Gimap3: A Foot-in-the-Door to Tissue-Specific Regulation of Mitochondrial DNA Genetics. Small GTPases (2011) 2(1):31–5. 10.4161/sgtp.2.1.14937 PMC3116618 21686279

[126] Jenuth JP Peterson AC Fu K Shoubridge EA. Random Genetic Drift in the Female Germline Explains the Rapid Segregation of Mammalian Mitochondrial DNA. Nat Genet (1996) 14(2):146–51. 10.1038/ng1096-146 8841183

[127] Battersby BJ Shoubridge EA. Selection of a mtDNA Sequence Variant in Hepatocytes of Heteroplasmic Mice is Not Due to Differences in Respiratory Chain Function or Efficiency of Replication. Hum Mol Genet (2001) 10(22):2469–79. 10.1093/hmg/10.22.2469 11709534

[128] Battersby BJ Loredo-Osti JC Shoubridge EA. Nuclear Genetic Control of Mitochondrial DNA Segregation. Nat Genet (2003) 33(2):183–6. 10.1038/ng1073 12539044

[129] Battersby BJ Redpath ME Shoubridge EA. Mitochondrial DNA Segregation in Hematopoietic Lineages Does Not Depend on MHC Presentation of Mitochondrially Encoded Peptides. Hum Mol Genet (2005) 14(17):2587–94. 10.1093/hmg/ddi293 16049030

[130] Simpson JC Joggerst B Laketa V Verissimo F Cetin C Erfle H. Genome-Wide RNAi Screening Identifies Human Proteins With a Regulatory Function in the Early Secretory Pathway. Nat Cell Biol (2012) 14(7):764–74. 10.1038/ncb2510 22660414

[131] Shah DK Zuniga-Pflucker JC. An Overview of the Intrathymic Intricacies of T Cell Development. J Immunol (2014) 192(9):4017–23. 10.4049/jimmunol.1302259 24748636

[132] von Boehmer H. Unique Features of the Pre-T-Cell Receptor Alpha-Chain: Not Just a Surrogate. Nat Rev Immunol (2005) 5(7):571–7. 10.1038/nri1636 15999096

[133] Belver L Ferrando A. The Genetics and Mechanisms of T Cell Acute Lymphoblastic Leukaemia. Nat Rev Cancer (2016) 16(8):494–507. 10.1038/nrc.2016.63 27451956

[134] Girardi T Vicente C Cools J De Keersmaecker K. The Genetics and Molecular Biology of T-ALL. Blood (2017) 129(9):1113–23. 10.1182/blood-2016-10-706465 PMC5363819 28115373

[135] Aifantis I Raetz E Buonamici S. Molecular Pathogenesis of T-Cell Leukaemia and Lymphoma. Nat Rev Immunol (2008) 8(5):380–90. 10.1038/nri2304 18421304

[136] Teitell MA Pandolfi PP. Molecular Genetics of Acute Lymphoblastic Leukemia. Annu Rev Pathol (2009) 4:175–98. 10.1146/annurev.pathol.4.110807.092227 18783329

[137] Zhong Y Jiang L Hiai H Toyokuni S Yamada Y. Overexpression of a Transcription Factor LYL1 Induces T- and B-Cell Lymphoma in Mice. Oncogene (2007) 26(48):6937–47. 10.1038/sj.onc.1210494 17486074

[138] Hsu HL Wadman I Baer R. Formation of In Vivo Complexes Between the TAL1 and E2A Polypeptides of Leukemic T Cells. Proc Natl Acad Sci U S A (1994) 91(8):3181–5. 10.1073/pnas.91.8.3181 PMC43539 8159721

[139] Curtis DJ Robb L Strasser A Begley CG. The CD2-scl Transgene Alters the Phenotype and Frequency of T-lymphomas in N-ras Transgenic or p53 Deficient Mice. Oncogene (1997) 15(24):2975–83. 10.1038/sj.onc.1201467 9416841

[140] Condorelli GL Facchiano F Valtieri M Proietti E Vitelli L Lulli V. T-Cell-Directed TAL-1 Expression Induces T-Cell Malignancies in Transgenic Mice. Cancer Res (1996) 56(22):5113–9. 8912842

[141] Dadi S Le Noir S Payet-Bornet D Lhermitte L Zacarias-Cabeza J Bergeron J. TLX Homeodomain Oncogenes Mediate T Cell Maturation Arrest in T-ALL Via Interaction With ETS1 and Suppression of TCRalpha Gene Expression. Cancer Cell (2012) 21(4):563–76. 10.1016/j.ccr.2012.02.013 22516263

[142] Chadwick N Zeef L Portillo V Fennessy C Warrander F Hoyle S. Identification of Novel Notch Target Genes in T Cell Leukaemia. Mol Cancer (2009) 8:35. 10.1186/1476-4598-8-35 19508709 PMC2698846

[143] Chadwick N Zeef L Portillo V Boros J Hoyle S van Doesburg JC. Notch Protection Against Apoptosis in T-ALL Cells Mediated by GIMAP5. Blood Cells Mol Dis (2010) 45(3):201–9. 10.1016/j.bcmd.2010.07.006 20817506

[144] Wang H Zou J Zhao B Johannsen E Ashworth T Wong H. Genome-Wide Analysis Reveals Conserved and Divergent Features of Notch1/RBPJ Binding in Human and Murine T-Lymphoblastic Leukemia Cells. Proc Natl Acad Sci U S A (2011) 108(36):14908–13. 10.1073/pnas.1109023108 PMC3169118 21737748

[145] Liau WS Tan SH Ngoc PCT Wang CQ Tergaonkar V Feng H. Aberrant Activation of the GIMAP Enhancer by Oncogenic Transcription Factors in T-Cell Acute Lymphoblastic Leukemia. Leukemia (2017) 31(8):1798–807. 10.1038/leu.2016.392 PMC5529293 28028313

[146] Eckerle S Brune V Doring C Tiacci E Bohle V Sundstrom C. Gene Expression Profiling of Isolated Tumour Cells From Anaplastic Large Cell Lymphomas: Insights Into its Cellular Origin, Pathogenesis and Relation to Hodgkin Lymphoma. Leukemia (2009) 23(11):2129–38. 10.1038/leu.2009.161 19657361

[147] Berg HE Blackburn PR Baughn LB Ketterling RP Xu X Greipp PT. Identification of a Novel KMT2A/GIMAP8 Gene Fusion in a Pediatric Patient With Acute Undifferentiated Leukemia. Genes Chromosomes Cancer (2021) 60(2):108–11. 10.1002/gcc.22902 33078871

[148] Kannan K Kaminski N Rechavi G Jakob-Hirsch J Amariglio N Givol D. DNA Microarray Analysis of Genes Involved in p53 Mediated Apoptosis: Activation of Apaf-1. Oncogene (2001) 20(26):3449–55. 10.1038/sj.onc.1204446 11423996

[149] Milan M Dalla Rovere G Smits M Ferraresso S Pastore P Marin MG. Ecotoxicological Effects of the Herbicide Glyphosate in Non-Target Aquatic Species: Transcriptional Responses in the Mussel Mytilus Galloprovincialis. Environ Pollut (2018) 237:442–51. 10.1016/j.envpol.2018.02.049 29505984

[150] McDowell IC Modak TH Lane CE Gomez-Chiarri M. Multi-Species Protein Similarity Clustering Reveals Novel Expanded Immune Gene Families in the Eastern Oyster Crassostrea Virginica. Fish Shellfish Immunol (2016) 53:13–23. 10.1016/j.fsi.2016.03.157 27033806

[151] Balla KM Rice MC Gagnon JA Elde NC. Linking Virus Discovery to Immune Responses Visualized During Zebrafish Infections. Curr Biol (2020) 30(11):2092–103.e5. 10.1016/j.cub.2020.04.031 32413307 PMC7854381

[152] Ye X Zhou L Jia J Wei L Wen Y Yan X. ITRAQ Proteomic Analysis of Yellow and Black Skin in Jinbian Carp (Cyprinus Carpio). Life (Basel) (2020) 10(10):1–19. 10.3390/life10100226 PMC7601221 33007994

[153] Weirich CS Erzberger JP Barral Y. The Septin Family of GTPases: Architecture and Dynamics. Nat Rev Mol Cell Biol (2008) 9(6):478–89. 10.1038/nrm2407 18478031

